# Alcohol Dehydrogenases with *anti*‐Prelog Stereopreference in Synthesis of Enantiopure Alcohols

**DOI:** 10.1002/open.202100251

**Published:** 2022-02-22

**Authors:** Musa M. Musa

**Affiliations:** ^1^ Department of Chemistry Interdisciplinary Research Center for Refining and Advanced Chemicals King Fahd University of Petroleum and Minerals Dhahran 31261 Saudi Arabia

**Keywords:** alcohol dehydrogenases, *anti*-Prelog, asymmetric reduction, biocatalysis, enantiopure alcohols

## Abstract

Biocatalytic production of both enantiomers of optically active alcohols with high enantiopurities is of great interest in industry. Alcohol dehydrogenases (ADHs) represent an important class of enzymes that could be used as catalysts to produce optically active alcohols from their corresponding prochiral ketones. This review covers examples of the synthesis of optically active alcohols using ADHs that exhibit *anti*‐Prelog stereopreference. Both wild‐type and engineered ADHs that exhibit *anti*‐Prelog stereopreference are highlighted.

## Graphical abstract

**Alcohol Dehydrogenases (ADHs) with** ***anti*** **‐Prelog stereopreference**, both wild‐type and engineered, can be used for asymmetric reductions of prochiral ketones. Findings and insights that might guide researchers to construct more ADHs with *anti*‐Prelog stereopreference are critically reviewed in this contribution.

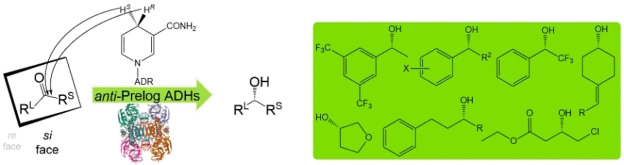

## 1 Introduction

Optically active alcohols are important building blocks of compounds that can be used in the agrochemical, flavor, pharmaceutical, and food industries.^[1]^ It is highly desirable to develop enantiopure drugs, or show sufficient evidence that the undesired enantiomer is not toxic.^[2]^ Biocatalytic transformations represent an attractive approach to synthesize enantiopure alcohols on an industrial scale. Biocatalysts are known for their high chemo‐, regio‐, and stereoselectivities; moreover, biocatalysis adheres to 10 of the 12 principles of green chemistry.[^3^, ^4^, ^5^] Although lipase‐catalyzed kinetic resolution is heavily used in industry,[^6^, ^7^, ^8^, ^9^, ^10^] the fact that optically active alcohols can be obtained with a maximum yield of 50 % with high enantiopurity represents a major drawback for this approach. Deracemization, which includes obtaining enantiopure compounds from their racemates in as high as 100 % yield, is an attractive strategy that has gained interest in the last two decades,^[11]^ but the fact that two or more catalysts are required to simultaneously operate in the same vessel restricts the industrial applications of the reported deracemization approaches. Deracemization is a thermodynamically uphill process.^[12]^ Thus, asymmetric reduction represents an attractive approach that can be used to synthesize optically active alcohols in high yields and high enantioselectivities.

**Figure 1:**
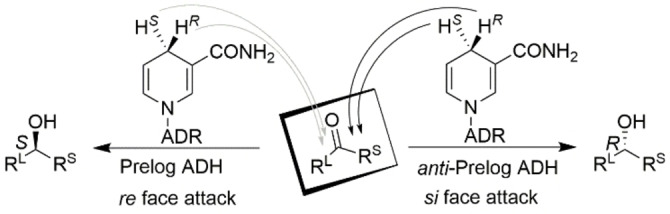
Stereochemical outcome of ADH‐catalyzed asymmetric transformations. R^L^ is more sterically hindered and exhibits higher Cahn‐Ingold‐Prelog priority than R^S^ (ADR: adenine diribose).

Alcohol dehydrogenases (ADHs, EC 1.1.1.X, X=1 or 2), which catalyze the interconversion of alcohols and their corresponding aldehydes or ketones, represent an important class of enzymes that can be used in the synthesis of optically active alcohols.^[13]^ ADHs require the use of a cofactor, which is either the nicotinamide‐adenine dinucleotide (NAD^+^) or its phosphate ester (NADP^+^). In an ADH‐catalyzed asymmetric reduction of prochiral ketones, there are four possibilities for delivering the hydride of the cofactor to the substrate. The pro‐(*R*) or pro‐(*S*) hydride of the cofactor can be delivered from either the *re* face or the *si* face of a prochiral ketone (Figure 1). Prelog used a diamond lattice mnemonic device to predict the stereoselectivity of ADH‐catalyzed asymmetric reduction of prochiral ketones.^[14]^ The majority of ADHs follow Prelog's rule in which the cofactor delivers its hydride from the *re* face, producing (*S*)‐alcohols when the large substituent of the ketone exhibits a higher Cahn–Ingold–Prelog (CIP) priority than that for the smaller substituent; note that, on some occasions, (*R*)‐alcohols are produced using *re*‐facial attack because the smaller substituent has a higher CIP priority.

Alcohol dehydrogenases that exhibit *anti*‐Prelog stereopreference are rare, and do not satisfy the huge demand for production of optically active alcohols using asymmetric reduction of prochiral ketones with *anti*‐Prelog stereopreference. Thus, the discovery of more ADHs with this stereopreference, be the either wild‐type or engineered enzymes, is crucial. This review highlights the efforts devoted to discover ADHs with *anti*‐Prelog stereopreference.

## 2 Discussion

### 2.1 Wild‐type ADHs with anti‐Prelog Stereopreference

**Scheme 1:**
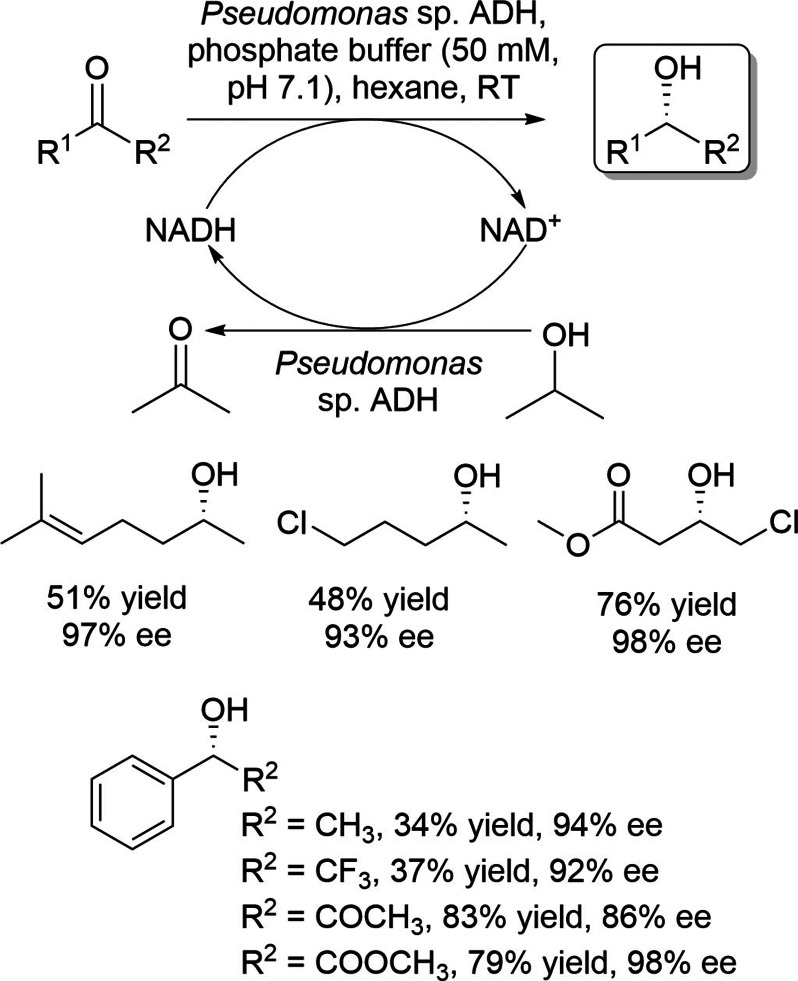
Asymmetric reduction of prochiral ketones using *Pseudomonas* sp. ADH.

**Scheme 2:**
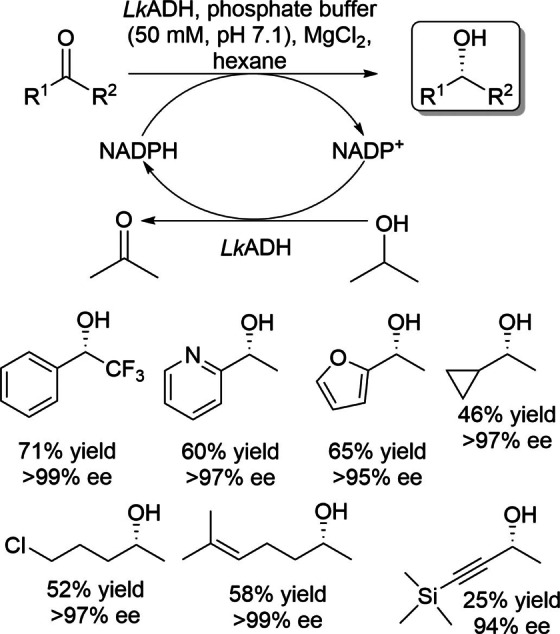
Asymmetric reduction of prochiral ketones using *Lactobacillus kefir* ADH.

**Scheme 3:**
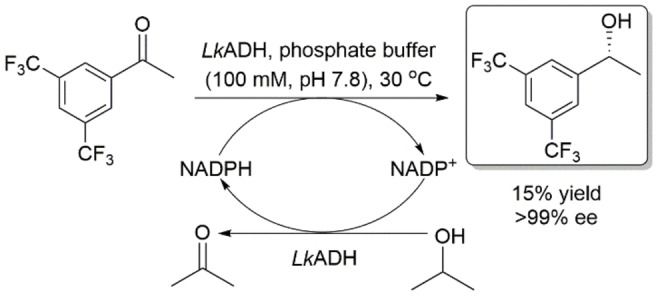
Asymmetric reduction of 1‐[3,5‐bis(trifluoromethyl)phenyl]ethanone using *Lactobacillus kefir* ADH.

This section describes wild‐type ADHs, or carbonyl reductases, which exhibit *anti*‐Prelog stereopreference in the asymmetric reduction of prochiral ketones. Wong and coworkers reported the isolation and characterization of an ADH from *Pseudomonas* sp., an NAD^+^‐dependent ADH which shows *anti*‐Prelog stereopreference in the asymmetric reduction of a wide variety of prochiral ketones (Scheme 1).[^15^, ^16^] They applied a substrate‐coupled approach using 2‐propanol for cofactor regeneration. The same research group reported the asymmetric reduction of prochiral ketones using ADH from *Lactobacillus kefir* (*Lk*ADH), an NADP^+^‐dependent ADH,^[17]^ which was reported earlier by Hummel.[^18^, ^19^] This enzyme showed high stereoselectivity in the asymmetric reduction of various prochiral ketones in *anti*‐Prelog mode (Scheme 2). Nuclear magnetic resonance (NMR) analysis of the transfer of deuteride from propanol‐d_8_ indicates that these enzymes deliver the pro‐(*R*) hydride of their cofactors from the *si* face of prochiral ketones (i. e., *anti*‐Prelog mode). *Lk*ADH was then cloned and expressed in *Escherichia coli* and the recombinant enzyme was used at preparative scale.^[20]^ Prochiral ketones were reduced with excellent enantioselectivities (>99 % ee) using the purified enzyme and glucose dehydrogenase for cofactor regeneration. (*R*)‐1‐[3,5‐Bis(trifluoromethyl)phenyl]‐ethanol, which is a building block of Aprepitant used in the treatment of chemotherapy‐induced nausea and vomiting, was also produced in excellent enantiopurity and low yield by the asymmetric reduction of its prochiral ketone using *L. kefir* (Scheme 3).^[21]^

**Scheme 4:**
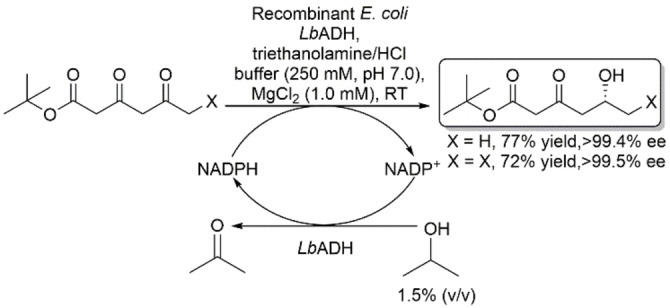
Asymmetric reduction of β,δ‐diketo esters using *Lactobacillus brevis* ADH.

Alcohol dehydrogenase from *Lactobacillus brevis* (*Lb*ADH) is a robust inserting *anti*‐Prelog NADP^+^‐dependent ADH that exhibits a wide substrate scope of alkyl and aryl ketones.^[22]^ This enzyme operates using a coupled‐substrate regeneration approach for NADPH that utilizes 2‐propanol, first explained by Hummel.^[23]^ Müller and coworkers reported the asymmetric reduction of 3,5‐dioxocarboxylates using recombinant *E. coli* expressing *Lb*ADH (Scheme 4).^[24]^ *tert*‐Butyl 3,5‐dioxohexanoate was reduced in high regio‐ and enantioselectivity to the corresponding (*R*)‐5‐hydroxy‐3‐oxohexanoate in 77 % yield and >99 % ee using this method. (*S*)‐6‐Chloro‐5‐hydroxy‐3‐oxohexanoate, which exhibits CIP priorities that do not match the steric demand of the substituents (i. e., the smaller group exhibits higher CIP than the larger group), was also produced in a similar yield and regio‐ and enantioselectivities. Such high regio‐ and enantioselectivity is not easily obtained using organic or organometallic catalysts.

**Scheme 5:**
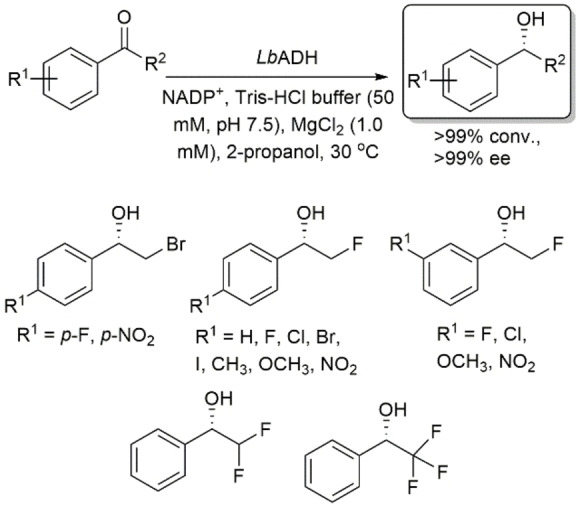
Asymmetric reduction of α‐halogenated ketones using *Lactobacillus brevis* ADH.

Gotor and coworkers reported the asymmetric reduction of a range of phenyl‐ring‐containing α‐halogenated ketones using *Lb*ADH, and their corresponding (*S*)‐halohydrins were quantitatively produced in >99 % ee in all cases (Scheme 5).^[25]^ These reactions showed similar conversions and enantioselectivities when conducted at substrate concentrations of 30 mm or 0.5 m, and due to the activated nature of these substrates, a substantial excess of the hydrogen donor was not required when using 0.5 m of substrate.

**Scheme 6:**
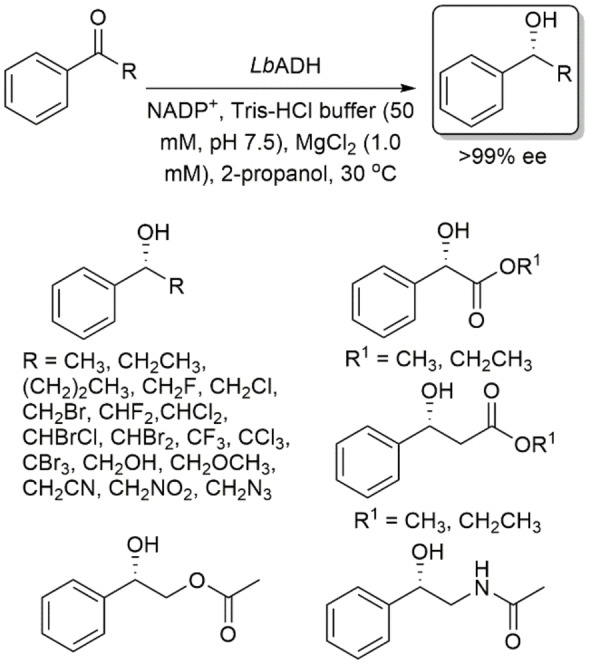
Asymmetric reduction of acetophenone derivatives bearing substituents that vary in size using *Lactobacillus brevis* ADH.

Using *Lb*ADH, Lavandera, Gotor and coworkers studied the asymmetric reduction of a variety of acetophenone derivatives bearing substituents that varied in size and electronic properties.^[26]^ Their results indicated that this enzyme accepts acetophenone derivatives not only with small groups such as methyl, ethyl or halomethyl, but also with more sterically hindered groups such as α,α,α‐trichloromethyl and α‐ethoxycarbonyl (Scheme 6). They found that the activity of the enzyme is dependent on the size of the substituent; however, electronic factors cannot be ignored.

**Scheme 7:**
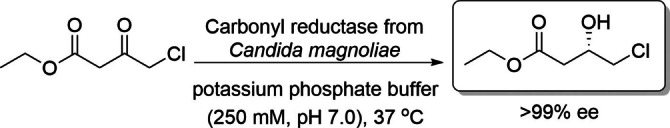
Asymmetric reduction of ethyl 4‐chloro‐3‐oxobutanoate using carbonyl reductase from *Candida magnoliae*.

Wada et al. reported the characterization and purification of a carbonyl reductase from *Candida magnoliae*, an NADP^+^‐dependent ADH.^[27]^ They showed that this enzyme reduces ethyl 4‐chloro‐3‐oxobutanoate (COBE) to produce ethyl (*S*)‐4‐chloro‐3‐hydroxybutanoate [(*S*)‐CHBE], the *anti*‐Prelog product, in excellent enantioselectivity (Scheme 7). (*S*)‐CHBE is a building block of hydroxymethylglutaryl‐CoA reductase inhibitors such as Lipitor, which are cholesterol‐lowering drugs. Moreover, they showed that this enzyme reduces conjugated diketones as well as α‐ and β‐ketoesters. It is worth mentioning that asymmetric reduction of COBE using whole cells of *Candida magnoliae* produced (*S*)‐CHBE in a lower enantioselectivity than that using the purified carbonyl reductase, which is attributed to the presence of other ADHs that exhibit various extents of stereoselectivity or opposite stereopreference in the cells. Xu and coworkers reported an NADH‐dependent carbonyl reductase from *Streptomyces coelicolor* that is capable of producing (*S*)‐CHBE in high yield (93 %), excellent enantiopurity (>99 % ee), and in high productivity (as high as 600 g L^−1^ in 22 h) in a biphasic water/toluene system.^[28]^

**Scheme 8:**
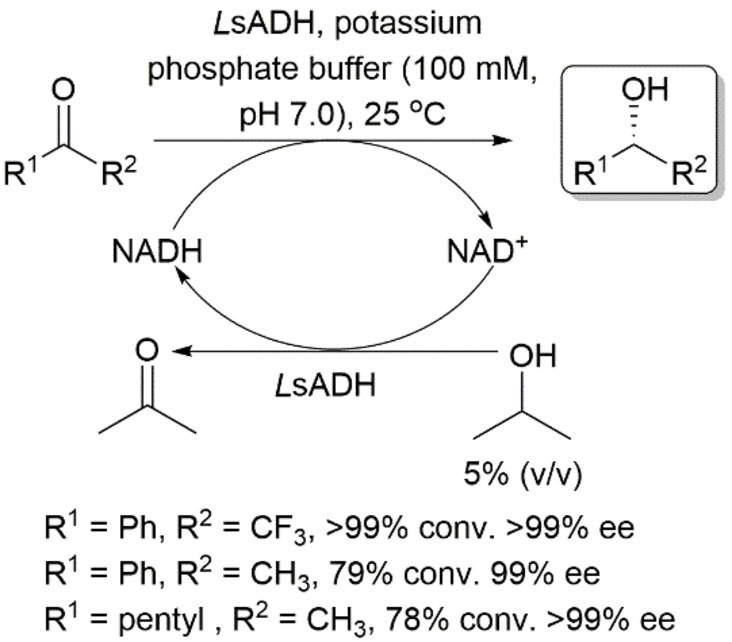
Asymmetric reduction of prochiral ketones using *Leifsonia* sp. ADH (*Ls*ADH) in *anti*‐Prelog mode.

Itoh and coworkers reported the asymmetric reduction of ketones using *Leifsonia* sp. ADH (*L*sADH), an NAD^+^‐dependent ADH.^[29]^ This enzyme was shown to reduce acetophenone and 2‐heptanone to their corresponding (*R*)‐alcohols and phenyl trifluoromethyl ketone to its corresponding (*S*)‐1‐phenyl‐2,2,2‐trifluoroethanol in very high enantioselectivities and good yields (Scheme 8). Utilization of a substrate‐coupled approach using 2‐propanol was used to regenerate the cofactor.

**Scheme 9:**
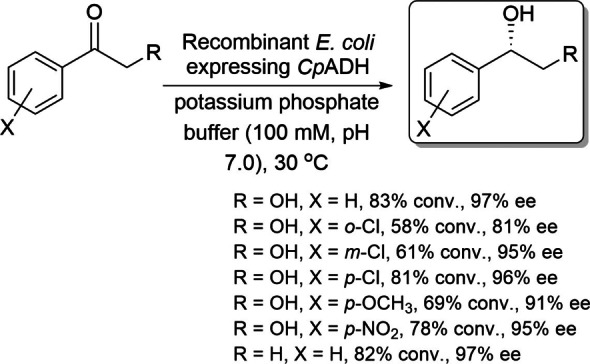
*anti*‐Prelog synthesis of aryl alcohols using recombinant *E. coli* expressing *Cp*ADH.

Xu and coworkers reported the ADH from *Candida parapsilosis* (*Cp*ADH), an NADP^+^‐dependent ADH, and showed that it reduces 2‐hydroxyacetophenone with *anti*‐Prelog stereopreference to produce (*S*)‐1‐phenyl‐1,2‐ethanediol with high enantiopurity (>99 % ee).[^30^, ^31^] The same group then reported the use of a recombinant *E. coli* system expressing *Cp*ADH in the asymmetric reduction of 2‐hydroxyacetophenone analogs.^[32]^ (*S*)‐1‐Phenyl‐1,2‐ethanediols were produced using this method with moderate yields and high enantioselectivities (Scheme 9). Moreover, three carbonyl reductases from *C. parapsilosis* were also reported and shown to exhibit *anti*‐Prelog stereopreference.^[33]^

The crystal structure of *Cp*ADH was solved, indicating that it forms a homo‐tetramer with the entrance of its NADPH pocket blocked by a surface loop.^[34]^ A number of mutations were carried out to confirm the importance of the catalytic triad Ser‐Tyr‐Lys, and the double point mutation of the amino acids that line the coenzyme‐binding pocket (S67D/H68D) resulted in a significant increase in preference towards NADH and a decreased preference to NADPH.

**Scheme 10:**
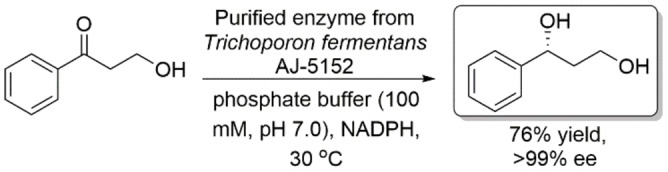
Asymmetric reduction of 3‐hydroxy‐1‐phenylpropane‐1‐one using a purified enzyme from *Trichoporon fermentans*, AJ‐5152.

Kira and Onishi reported an NADPH‐dependent enzyme from *Trichoporon fermentans*, AJ‐5152, that is capable of reducing 3‐hydroxy‐1‐phenylpropane‐1‐one to produce (*R*)‐1‐phenyl‐1,3‐propanediol with excellent enantioselectivity (Scheme 10).^[35]^ A yield of 8.9 g L^−1^ was produced in 16 h by successive feeding of substrate.

Meng and Xu reported an *anti*‐Prelog ADH from *Oenococcus oeni*, an NADP^+^‐dependent enzyme, which exhibits activity for 2‐octanone, 2‐hexanone, 2‐heptanone, and acetophenone.^[36]^ Zheng and coworkers reported an NADP^+^‐dependent *anti*‐Prelog short‐chain ADH from *Lactobacillus composti*.^[37]^ This enzyme was expressed in *E. coli* and used in the reduction of acetophenone to (*R*)‐1‐phenylethanol using glucose dehydrogenase for cofactor regeneration.

Lopez‐Gallego and coworkers reported a short chain ADH from *Thermus thermophilus* HB27, which is the first report of a thermophilic ADH that exhibits *anti*‐Prelog stereopreference.^[38]^ This enzyme showed better activity when NADH was used as the cofactor in comparison with NADPH. It was active towards a wide variety of ketones and aldehydes, as well as their alcohols and β‐hydroxyesters. Asymmetric reductions of 2‐phenylpropionaldehyde and 2,2,2‐trifluoroacetophenone using the enzyme immobilized on an agarose mixture resulted in formation of (*R*)‐2‐phenyl‐1‐propanol and (*S*)‐1‐phenyl‐2,2,2‐trifluoroethanol with enantioselectivities of 71 % and 96 % ee, respectively.

**Scheme 11:**
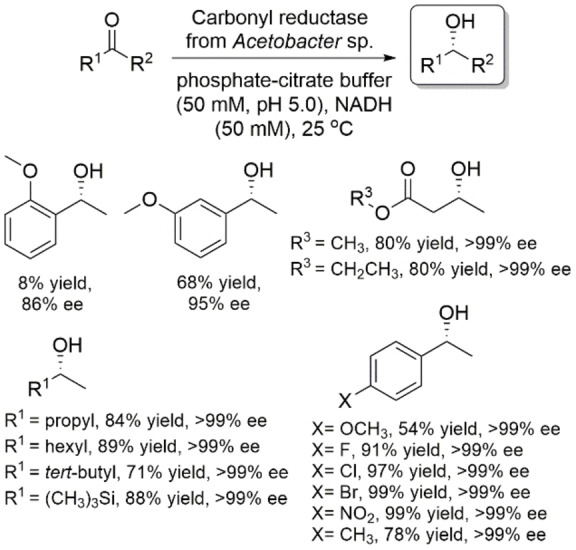
Asymmetric reduction of various ketones using carbonyl reductase from *Acetobacter* sp. CCTCC M209061.

Lou and coworkers reported a carbonyl reductase from *Acetobacter* sp. CCTCC M209061 that exhibited *anti*‐Prelog stereopreference in the asymmetric reduction of prochiral ketones (Scheme 11).^[39]^ This enzyme accepted a large range of substrates including acetophenone analogs, α‐ketoesters and aliphatic ketones. It also showed a preference for NADH when compared with NADPH.

Wu and coworkers reported a ketone reductase from *Chryseobacterium* sp. CA49, which could reduce 3,5‐bis(trifluoromethyl)acetophenone in *anti*‐Prelog mode to produce the corresponding (*R*)‐configured alcohol with excellent yield and enantioselectivity.^[40]^ They used lyophilized powder of crude recombinant enzyme to generate (*R*)‐1‐[3,5‐bis‐(trifluoromethyl)phenyl]ethanol using the asymmetric reduction of the corresponding ketone at a scale of 150 g L^−1^ within 24 h using 2‐propanol as a cosubstrate, showcasing potential for industrial applications.

**Scheme 12:**
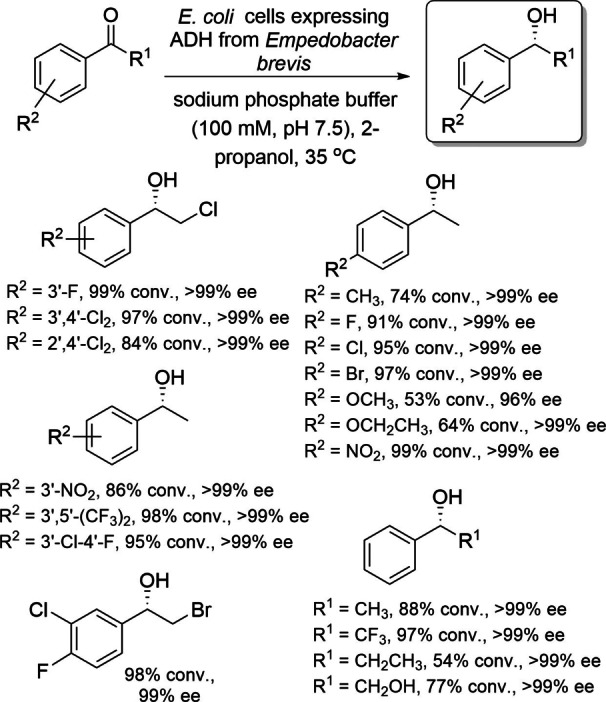
Asymmetric reduction of acetophenone analogs using *E. coli* whole cells expressing *Empedobacter brevis*.

Yu and coworkers reported an NAD^+^‐dependent short chain ADH from *Empedobacter brevis* that is capable of reducing prochiral acetophenone analogs in *anti*‐Prelog mode.^[41]^ Asymmetric reduction of acetophenones using *E. coli* whole cells expressing this ADH resulted in production of their optically active alcohols with good activity and excellent stereoselectivity. They had *anti*‐Prelog stereopreference and there was no requirement to add any quantity of the cofactor (Scheme 12).

**Scheme 13:**
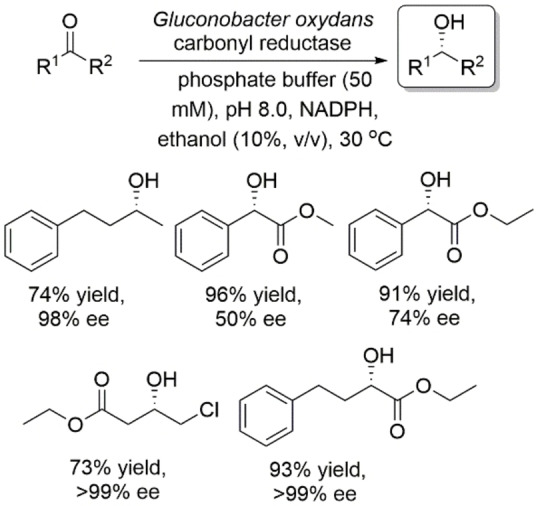
Asymmetric reduction of prochiral ketones using carbonyl reductase from *Gluconobacter oxydans*.

Lin, Wei and coworkers reported an NADP^+^‐dependent carbonyl reductase from *Gluconobacter oxydans* DSM2343.^[42]^ This enzyme reduced aliphatic and aromatic ketones as well as α‐ and β‐ketoesters to produce the corresponding optically active alcohols in high yields and moderate to excellent enantioselectivities with *anti*‐Prelog mode (Scheme 13). Docking studies of COBE with enzyme‐cosubstrate indicate that the hydride of NADPH is delivered from the *si* face of the prochiral ketones.

**Scheme 14:**
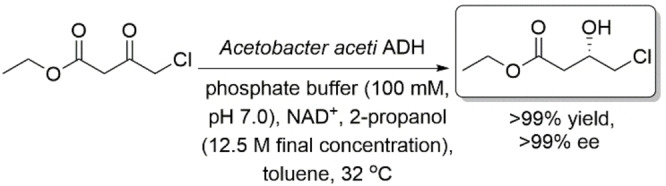
Asymmetric reduction of ethyl 4‐chloro‐3‐oxobutanoate using *Acetobacter aceti* ADH.

Wangikar and coworkers identified ADHs that exhibit *anti*‐Prelog stereopreference. They found that the ADH from *Acetobacter aceti*, an NAD^+^‐dependent ADH, exhibits high specific activities on β‐ketoesters.^[43]^ More specifically, using lyophilized cell‐free extract of this ADH allowed the asymmetric reduction of COBE to produce (*S*)‐CHBE, the *anti*‐Prelog product, in excellent yield and enantioselectivity (Scheme 14). This enzyme also showed good activities with other β‐ketoesters such as methyl 4‐chloro‐3‐oxo‐butanoate, ethyl 3‐oxobutanoate and methyl 3‐oxobutanoate.

Asymmetric reduction reactions of prochiral ketones in *anti*‐Prelog mode have been reported using whole cells of *Acetobacter pasteurianus* GIM1.158,^[44]^ *Leifsonia xyli* HS0904,^[45]^ *Leifsonia xyli* CCTCCM 2010241,^[46]^ *Lactobacillus reuteri* DSM 20016, ^[47]^ and *Penicillium citrinum* VIT SS2,^[48]^ and also when using the fungus *Penicillium expansum*.^[49]^ The use of whole cells is attractive for several reasons, including production cost, space‐time‐yield and easier procedures.[^50^, ^51^] However, the reaction mechanism is unclear because of the possibility of involvement of more than one enzyme in whole cell biocatalysis.

Reaction medium engineering using deep eutectic solvents was reported as another approach to switch the stereopreference from Prelog to *anti*‐Prelog in asymmetric reduction of prochiral ketones using whole cells.^[52]^ This was explained by the possibility of inhibiting Prelog ADHs when the reaction medium is changed, and thus paves the way for asymmetric reduction using *anti*‐Prelog ADH(s). Reaction medium engineering is a very attractive approach that enables tuning the reaction stereopreference, stereoselectivity and yield without the need for laborious directed evolution or site‐directed mutagenesis.[^53^, ^54^, ^55^] Asymmetric reduction using whole cells is beyond the scope of this review and therefore will not be discussed in detail here.

### 2.2 Engineered ADHs with anti‐Prelog Stereopreference

The number of ADHs that exhibit excellent stereoselectivity with *anti*‐Prelog stereopreference is limited. Thus, protein engineering using rational site‐directed mutagenesis and directed evolution along with molecular dynamics (MD) simulations represent a key approach to producing such enzymes.[^56^, ^57^, ^58^, ^59^] This section describes examples of ADH mutants that were designed and constructed to perform asymmetric reduction of prochiral ketones in *anti*‐Prelog mode.

Secondary ADH from *Thermoanaerobacter pseudoethanolicus* (*Te*SADH), a robust thermophilic enzyme, follows Prelog stereoselectivity.^[60]^ It exhibits two binding pockets that vary in size and affinity to the substituents of substrates. *Te*SADH is identical to the well‐known secondary ADH from *Thermoanaerobacter brockii* (*Tb*SADH).^[60]^ Phillips and coworkers reported a reversal in stereopreference of *Te*SADH from (*S*)‐ to (*R*)‐2‐pentanol upon the single point mutation in S39T *Te*SADH.^[61]^ In some cases, asymmetric reduction of substituted 2‐tetralones resulted in a switch in the stereopreference (i. e., production of *anti*‐Prelog 2‐tetralol analogs).[^62^, ^63^, ^64^] Docking studies indicate that the stereopreference of these reactions is controlled by the position of the substituent, which influences the binding orientation of these 2‐tetralones, and thus allows the delivery of hydride from the *si* face. Such substrate‐size‐induced reversal of stereopreference was also observed in the *Te*SADH‐catalyzed asymmetric reduction of ethynyl ketones that exhibit a short chain,[^65^, ^66^] and in the *Tb*SADH‐catalyzed asymmetric reduction of aliphatic ketones that exhibit short alkyl sites.^[67]^ This substrate‐size‐induced stereospecificity, which results in the formation of *anti*‐Prelog products in the asymmetric reduction of ketones that exhibit short chains, was explained by the higher affinity of the smaller pockets, when compared to the larger one, of the active site of *Tb*SADH and *Te*SADH towards alkyl groups.

**Scheme 15:**
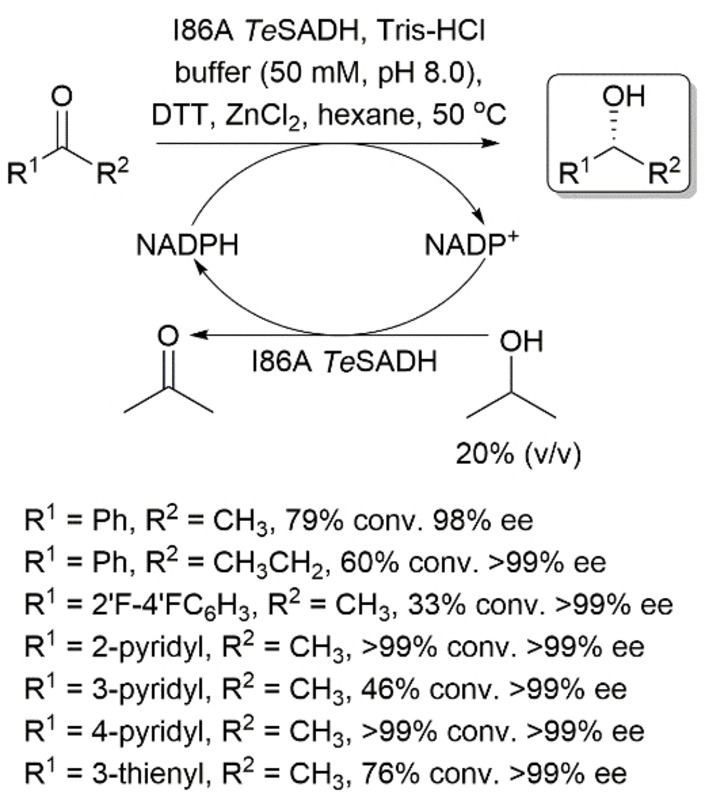
Asymmetric reduction of phenyl‐ring‐containing ketones using I86A *Te*SADH. DTT: Dithiothreitol.

Phillips and coworkers reported that a single point mutation at I86, which lines the small pocket in *Te*SADH, resulted in an *anti*‐Prelog *Te*SADH mutant (I86A) that accommodates acetophenone and its analogs and produces their corresponding (*R*)‐alcohols in high stereoselectivities and moderate to high yields (Scheme 15).^[68]^ Further expansion of the small pockets in I86A/C295A, A85G/I86A/C295A, I86A/V115A/C295A, and I86A/Y153A/C295A *Te*SADHs allowed for the reduction of substituted acetophenones, which are not substrates for I86A *Te*SADH, in *anti*‐Prelog mode.[^69^, ^70^]

**Scheme 16:**
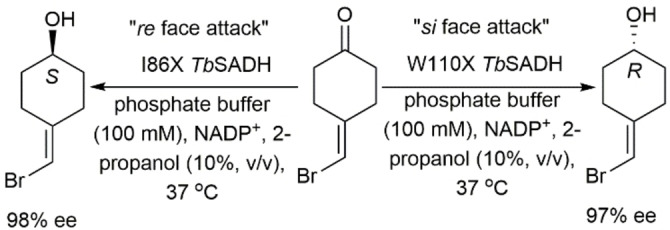
Asymmetric reduction of 4‐(bromomethylene)cyclohexanone using *Tb*SADH mutants that exhibit opposite stereopreferences.

**Figure 2:**
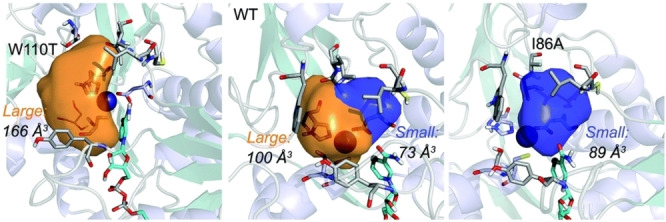
Shape and volume of the large and small binding pockets of WT *Tb*SADH, W110T, and I86A variants. Reproduced from Ref. [72], Copyright 2017, Royal Society of Chemistry.

Reetz and coworkers used combinatorial saturation mutagenesis with the *Tb*SADH crystal structure and found that, among the mutants tested, I86A *Tb*SADH reduced 4‐alkylidene cyclohexanones to their corresponding (*S*)‐alcohols in high stereoselectivity (Scheme 16). These cannot be asymmetrically reduced using transition metal catalysis.^[71]^ MD simulations using 4‐(bromomethylene)cyclohexanone as a substrate showed that the small pocket in *Tb*SADH was enlarged from 73 to 89 Å^3^ upon mutation of I86 to A (Figure 2),^[72]^ and that the C−H⋅⋅⋅π interactions between the indole ring of W110, which is part of the large binding pocket of the enzyme, and the cyclohexane ring of 4‐alkylidene cyclohexanones result in pro‐(*S*) hydride transfer from the *re* face of 4‐(bromomethylene)cyclohexanone. Moreover, they showed that the W110T mutation enlarged the large binding pocket in the enzyme from 100 to 166 Å^3^, which allows pro‐(*R*) hydride transfer to the *si* face of 4‐(bromomethylene)cyclohexanone, and thus producing the axially chiral (*R*)‐alcohols.

**Scheme 17:**
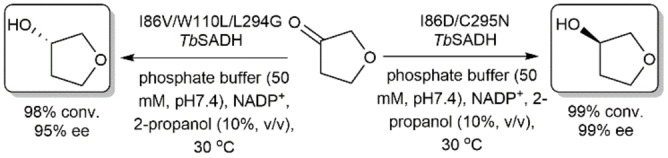
Asymmetric reduction of tetrahydrofuran‐3‐one using two variants of *Tb*SADH that exhibit opposite stereopreferences.

Reetz and coworkers used directed evolution utilizing the triple‐code saturation mutagenesis approach on *Tb*SADH to identify two variants that exhibit opposite stereopreferences in the asymmetric reduction of tetrahydrofuran‐3‐one and tertrahydrothiofuran‐3‐one.^[73]^ Asymmetric reduction of tetrahydrofuran‐3‐one using I86D/C295N and I86V/W110L/L294Q *Tb*SADHs produced (*R*)‐ and (*S*)‐3‐hydroxytetrahydrofuran, respectively, with high enantioselectivities (Scheme 17). Such high enantioselectivity is not easily obtained using organic or organometallic catalysts in asymmetric reduction of prochiral ketones bearing substituents that slightly vary in steric hindrance. (*S*)‐3‐Hydroxytetrahydrofuran is an important building block of Amprenavir and Fosamprenavir, which are HIV protease inhibitors.^[74]^ The above‐mentioned studies on *Te*SADH and *Tb*SADH clearly indicate the possibility to switch the stereopreference of these enzymes to enable the independent production of both enantiomers of alcohols with high enantioselectivities.

Kim and Plapp reported a switch in stereopreference when they studied the kinetics of stereospecific oxidation of secondary alcohols using the S48T/F93A mutant of horse liver alcohol dehydrogenase (HLADH).^[75]^ More specifically, they noticed a 10‐, 590‐, and 200‐fold increase in the enantiomeric ratio (*E* value=R/S) for 2‐butanol, 2‐octanol, and 1‐phenylethanol, respectively.

Xiao, Guo and coworkers solved the complex structures of *Cp*ADH with cofactor and substrate/product. They also studied the effect of mutation of F285 and W286 residues to alanine.^[76]^ These residues line the small binding pocket of *Cp*ADH and were selected because they are unique in *Cp*ADH and not conserved in other medium‐chain ADHs. They concluded that residues F285 and W286 might contribute to the pocket shape and orientation of the substrate, and thus play a central role in controlling the stereopreference and stereoselectivity of similar ADHs. This could help in designing more ADH mutants that exhibit *anti*‐Prelog stereopreference.

**Scheme 18:**
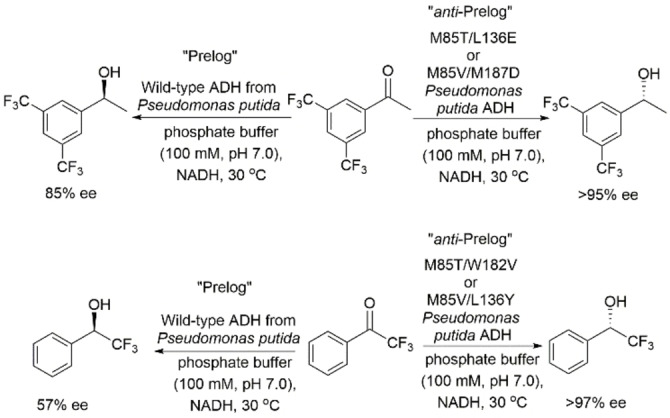
Asymmetric reduction of halogenated acetophenone analogs using *Pseudomonas putida* ADH (wild type) and using various double mutants.

Yu and coworkers reported an inversion in stereopreference of *Pseudomonas putida* ADH from Prelog to *anti*‐Prelog in the asymmetric reduction of halogenated acetophenones.^[77]^ Their study was guided by structural comparison with *Empedobacter brevis* ADH, an *anti*‐Prelog ADH, which led to identification of M85, L136, W182, and M187 as amino acids that could control the Prelog stereorecognition in *P. putida* ADH. Single and double point mutations at these sites resulted in *P. putida* ADH mutants that exhibit *anti*‐Prelog stereopreference. For instance, the double mutants M85T/L136E and M85V/M187D resulted in complete reversal in the stereopreference of the asymmetric reduction of 3′,5′‐bis(trifluoromethyl)acetophenone when compared to the wild‐type enzyme (Scheme 18). Docking studies of 3′‐chloro‐4′‐fluoroacetophenone in the wild‐type enzyme and M85T/L136E and M85V/M187D mutants indicated that halogen atoms and aromatic ring of the ketone substrate play a critical role in determining the stereopreference of these reactions. More specifically, *anti*‐Prelog stereopreference resulted from the anion⋅⋅⋅π interactions between the negatively charged residues E and D and the aromatic ring of the substrate, and from the hydrogen bonding between these residues and the halogen atoms of the substrate.

**Scheme 19:**
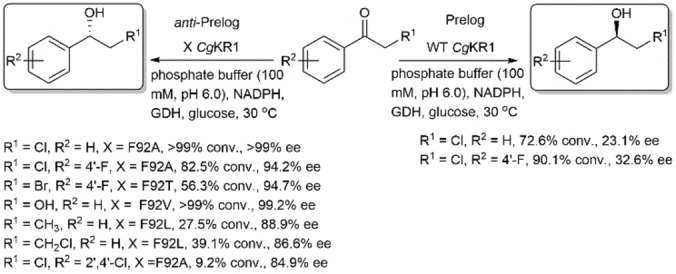
Asymmetric reduction of prochiral ketones using F92 mutants of *Candida glabrata* ketoreductase 1. GDH: glucose dehydrogenase.

You and coworkers reported a switch in the stereopreference of *Candida glabrata* ketoreductase 1 (*Cb*KR1) from Prelog to *anti*‐Prelog in the asymmetric reduction of α–haloketones to produce the corresponding (*S*)‐aryl‐halohydrins.^[78]^ They solved the crystal structure of *Cb*KR1 and then conducted docking studies of 2‐chloro‐acetophenone in the active site of the enzyme. This indicated that the chloromethyl group of the substrate is placed in a small binding pocket consisting of F92, F94, I172, and Y175, and the phenyl group of the substrate is accommodated in a large binding pocket that consists of S134, F135, A136, P206, V207, and Y208. This leads to pro‐*R* orientation of this substrate, which produces the Prelog product. Mutation of F92 with a less sterically hindered amino acid such as A, L, V, or T enlarges the small binding pocket, and allows the phenyl ring of the substrate to fit in the smaller pocket of *Cb*KR1, thus reducing prochiral ketones in *anti*‐Prelog mode (Scheme 19).

**Scheme 20:**
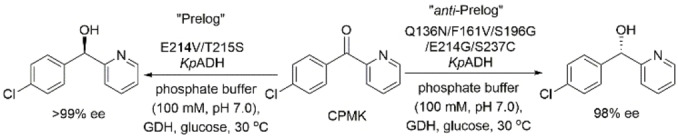
Switching the stereopreference of *Kluyveromyces polyspora* ADH in asymmetric reduction of aryl ketones.

Zhou, Ni and coworkers reported an inversion in the stereopreference of *Kluyveromyces polyspora* ADH (*Kp*ADH), an NADP^+^‐dependent Prelog ADH,^[79]^ in the asymmetric reduction of (4‐chlorophenyl)‐(pyridine‐2‐yl)ketone (CPMK).^[80]^ They identified the key residues inside and at the entrance of the substrate‐binding pocket using a polarity scanning strategy. They then used iterative combinatorial mutagenesis and showed that the variant Q136N/F161V/S196G/E214G/S237C *Kp*ADH produces (*S*)‐(4‐chlorophenyl)‐(pyridin‐2‐yl)methanol, the *anti*‐Prelog product, in 98 % ee from CPMK, and that the variant E214V/T215S *Kp*ADH produces the Prelog (*R*)‐alcohol in >99 % ee (Scheme 20). Subsequently, Sun and coworkers reported A85G/I86A/Q101A *Tb*SADH, which was identified using combinatorial active‐site double‐code saturation mutagenesis at sites that line the substrate‐binding pocket (Q101, W110, L294, and C295) as a catalyst for asymmetric reduction of CPMK to its corresponding (*S*)‐alcohol in excellent enantioselectivity.^[81]^

## 3 Summary and Outlook

Synthesis of each of the enantiomers of chiral alcohols is of interest. Most of the known ADHs obey Prelog's rule in their asymmetric reduction of prochiral ketones. Continued search for *anti*‐Prelog ADHs is highly desirable to enable the production of both enantiomers of an alcohol with high enantioselectivities, and thus does not restrict this process to the production of Prelog's products. This review highlights important findings in the literature that might help directing researchers towards designing new ADHs with *anti*‐Prelog stereopreference. Recent advances in molecular biology make it easier to control various characteristics of enzymes including substrate specificity, stereoselectivity, and stereopreference. However, the enzyme stereo‐recognition is challenging, as it is not dependent only on the sizes of substrate substituents, but also on non‐bonding interactions. Proper understanding of the enzyme–substrate interaction should allow for the switch from known Prelog ADHs to *anti*‐Prelog ones. This can be accomplished by directed evolution or using rationalized site‐directed mutagenesis combined with molecular dynamics simulations. Efforts should be directed towards the discovery of robust ADHs that exhibit *anti*‐Prelog stereopreferences in addition to wide substrate scopes. Extensive studies of medium engineering of ADH‐catalyzed reactions should also be conducted for encapsulated ADHs, which might help in tuning enantioselectivity.

## Conflict of interest

The author declares no conflict of interest.

4

## Biographical Information

*Musa M. Musa received his Ph.D. from University of Georgia in 2007 and then carried out postdoctoral research at University of Minnesota. In 2009, he joined King Fahd University of Petroleum and Minerals (KFUPM) as a faculty member. He is currently an Associate Professor of Organic Chemistry at KFUPM. His research interests include employing enzymes in organic synthesis. More specifically, he is interested in alcohol dehydrogenase‐catalyzed racemization, deracemization, stereoinversion and dynamic kinetic resolution of alcohols using alcohol dehydrogenases*.

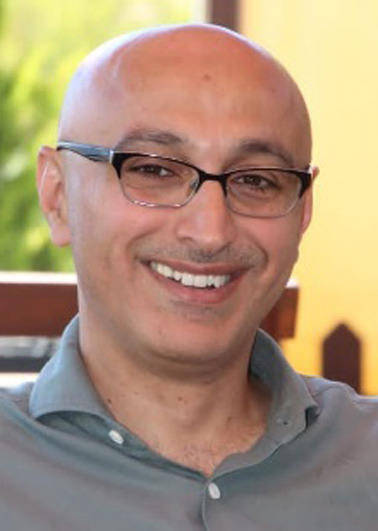

## Data Availability

Data sharing is not applicable to this article as no new data were created or analyzed in this study.
